# Emerging implications of bacterial biofilm in cancer biology: Recent updates and major perspectives

**DOI:** 10.1080/29933935.2024.2339270

**Published:** 2024-05-07

**Authors:** Manish Kushwaha, Vanditha Nukala, Akhilesh Kumar Singh, Govind K. Makharia, Anand Mohan, Anil Kumar, Nishu Dalal

**Affiliations:** a Gene Regulation Laboratory, National Institute of Immunology, New Delhi, India; b School of Life Science, Department of Biotechnology, Mahatma Gandhi Central University, Motihari, India; c Department of Biotechnology, School of Basic and Applied Sciences, Dayananda Sagar University, Bangalore, India; d Department of Gastroenterology and Human Nutrition, All India Institute of Medical Sciences, New Delhi, India; e School of Bioengineering and Biosciences, Lovely Professional University, Phagwara, India; f Azrieli Faculty of Medicine, Bar-Ilan University, Safed, Israel

**Keywords:** Biofilm, cancer biology, inflammation, bacteria, therapy

## Abstract

Recent insights have unveiled exciting opportunities to explore the intricate interplay among bacterial biofilms, tumor cells, and the immune system, thus offering new perspectives in cancer biology. The implications of bacterial biofilms in this context are remarkably multifaceted. Biofilms can promote tumor growth and invasiveness by inducing chronic inflammation, remodeling the extracellular matrix, and modulating the immune response, which promotes cancer development. Recent findings have demonstrated the involvement of distinct bacteria, like *Salmonella typhi* in gall bladder cancer, *Helicobacter pylori* in gastric cancer, and *Fusobacterium nucleatum* in oral cancer. These investigations indicate higher prevalence of these bacteria in individuals with cancer as compared to those who are healthy. Additionally, these bacteria create biofilms and display resistance to cancer treatments.In this review, we highlighted the recent advancements pertaining to influences of bacterial biofilm in cancer progression and potential molecular mechanisms by which bacterial biofilms contribute to cancer development.

## Introduction

Globally, cancer is one of the leading causes of death^1^ and has long been studied from genetics, tumor microenvironment, and immune response viewpoints. However, recent discoveries have highlighted a previously overlooked aspect of cancer biology: the presence and impact of bacterial biofilm in cancer causation and progression. Biofilm is defined as a collection of microorganisms embedded in a matrix of extracellular polymeric substances (EPS) and adhering to either living or non-living surfaces,^2–4^ has primarily been associated with chronic infections. The formation of stationary biofilm communities is a multifaceted and constantly changing process in which extracellular polymeric substances (EPS) play crucial roles in both structure and function, which are vital for the unique properties of biofilms as a whole. EPS facilitates the initial attachment of microbes to both living and non-living surfaces. Once adhered to, additional EPS production forms a matrix that envelops and binds the cells, maintaining their proximity and enabling interactions between them within a confined environment.^2^ The EPS matrix not only imparts mechanical stability but also creates intricate chemical microenvironments essential for the biofilm lifestyle.^5^

It should also be emphasized that biofilm-forming bacteria play a significant role in the development of numerous illnesses and infections that can be fatal in humans. These include cystic fibrosis (CF), otitis media, periodontitis, infective endocarditis (IE), chronic wounds, and osteomyelitis,^6–8^ Additionally, biofilms are implicated in vaginitis,^9^ colitis,^10^ conjunctivitis,^11^ gingivitis,^12^ and urethritis.^13^ It has been established that a substantial 80% of all bacterial infections in humans are connected to the formation of biofilms.^4,14^

Previous studies have shown that the primary effects of biofilm development in single bacterial models in vitro are drug resistance and environmental tolerance.^15,16^ Instead of being a stress response, biofilm development in the GI tract may be a proactive strategy. For instance, biofilm communities develop polybacterial structures and interspecies cross-feeding systems to promote co-colonization.^17^ Additionally, the finding of intestinal crypt biofilm and intracellular biofilm-like structures revealed that biofilms affect host cell function and cause chronic inflammation.^18–20^ Since biofilm development represents both invasion efficacy and persistence, it is appropriate to evaluate pathogenicity using this factor. However, their presence in various cancer types has raised intriguing questions about their role in tumorigenesis, cancer progression, treatment response, and clinical outcomes. Traditionally, cancer research has focused on intrinsic factors within cancer cells and their surrounding tissues. However, emerging evidence suggests that the microbial composition within the tumor microenvironment can significantly influence tumor behavior and treatment outcomes.^21^ Biofilms, known for their resistance to antibiotics and immune responses, exhibit similar characteristics in the context of cancer.^22^

**Figure 1.**
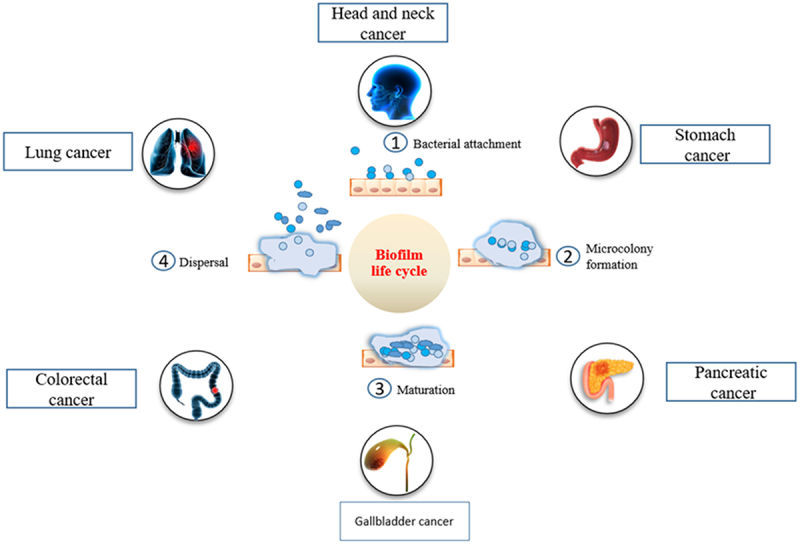
Association of bacterial biofilm with different types of cancers.

Bacterial biofilms have been identified in several cancer types, including colorectal, breast, pancreatic, and lung cancer, among others^23^ (Figure 1). They have been found within the tumor tissue, surrounding stromal cells, and distant metastatic sites. Several bacteria have been implicated in their potential roles in oncogenesis like *Helicobacter pylori*, *Salmonella typhi*, *Fusobacterium nucleatum*, *Parvimonas micra*, *Campylobacter jejuni*, *Chlamydia psittaci*, and *Chlamydia pneumonia* ^24^(Table 1). Various research reports have compellingly shown that biofilms play a significant role in the development of human colon cancer. Moreover, they have revealed a notable correlation between biofilms and the specific location of cancer within the colon. Notably, nearly all adenomas and cancers located on the right side of the colon are found to be associated with biofilms, underscoring their prominent role in this context. In contrast, left-sided colon cancers exhibit a notably lower prevalence of biofilm presence.^40^ Additionally, biofilms have also been implicated in the context of gastric cancer.^25^ When in their planktonic stage, these bacteria are only capable of causing a specific bacterial infection. It is only when they transition into the biofilm stage that they become associated with oncogenesis. Consequently, the presence of a certain group of bacteria within a biofilm may establish a distinct connection with the development of cancer. Bacteria could potentially be accountable for approximately 11% to 16% of global cancer cases, including gastrointestinal cancer.^41^ The development of cancer, known as oncogenesis, is not a single, instantaneous event but rather a complex process that necessitates the occurrence of multiple factors simultaneously.

**Table 1.** Possible role of bacteria in different types of cancers.

S⋅No	Cancer type	Bacteria	Mechanism	References
1.	Gastric Cancer	*Helicobacter pylori*	Induce the chronic inflammation	^25^
2.	Colorectal Cancer	*Fusobacterium nucleatum, Escherichia coli, Bacteroides fragilis*	Induce the chronic inflammation	^24,26,27^
3.	Gallbladder Cancer	*Salmonella typhi*	Induce the bacterial toxin and DNA damage	^28^
4.	Intestinal lymphomas	*Campylobacter jejuni*	Induce chronic inflammation and DNA damage	^24^
5.	Lung Cancer	*Chlamydia pneumonia, Mycobacterium tuberculosis*	Modulating Immune response	^24,29^
6.	Pancreatic cancer	*Porphyromonas gingivalis, Fusobacterium nucleatum*	Innate host defense and promotion of inflammation	^30,31^
7.	Colon cancer	*Streptococcus bovis*	Intestinal dysbiosis	^32,33^
8.	Esophageal cancer, Head and neck cancer	*Streptococcus anginosus*	Induce inflammation	^34^
9.	Oral Cancer	*Fusobacterium nucleatum*, *Porphyromonas gingivalis*	Induces genomic instability and promotes pro-tumor inflammation	^35^
10.	Salivary gland carcinoma	*Helicobacter pylori*, *Streptococcus* and *Rothia*	Induces genomic instability and promotes pro-tumor inflammation	^36,37^
11.	Pharyngeal cancer	*Prevotella and Streptococcus*	Induce inflammation and Intestinal dysbiosis	^38,39^

**Figure 2.**
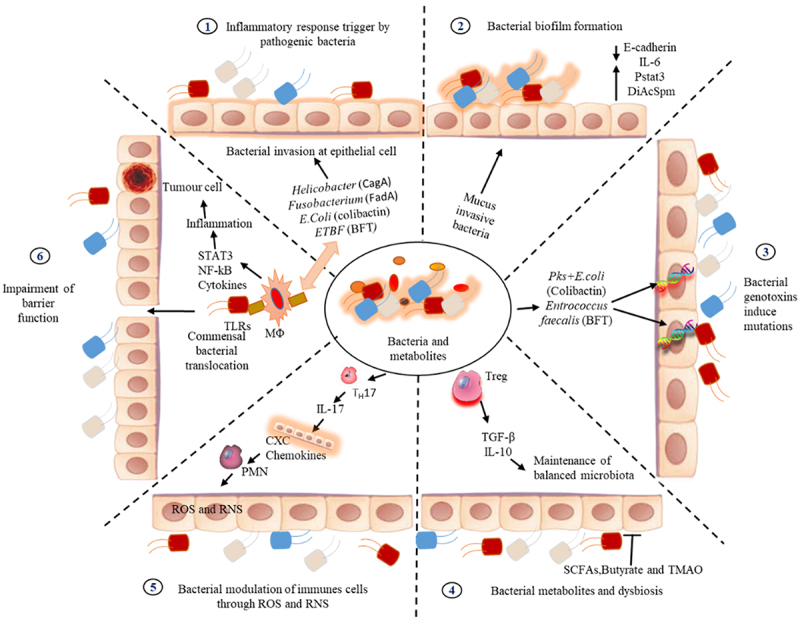
Mechanisms of cancer progression by bacterial biofilms. 1) Bacterial components trigger TLRs on tumor-infiltrating myeloid cells and macrophages, activating MyD88 and producing inflammatory cytokines like IL-23, TNF-α, and IL-6/11, amplifying NF-κB signalling, in myeloid cells, promoting tumour-associated inflammation 2) Bacterial biofilms in right-sided colorectal cancer (CRC) mucus are linked to early cancer-related changes in normal colon tissue, characterized by reduced E-cadherin, heightened STAT3 activation, increased epithelial IL-6, and elevated N1, N12-diacetylspermine (DiAcspm) levels, demonstrating carcinogenic potential of biofilm 3) Pathogenic bacteria, such as pks+ *Escherichia coli* and *B. fragilis*, triggering inflammation and tumorigenesis in normal colorectal tissues through the induction of mutations and genomic instability via microbial products like colibactin and *B. fragilis* toxin, serving as crucial initiators in colorectal cancer development 4) Commensal bacterial metabolites impact colorectal cancer(CRC) progression by influencing tumor dynamics and immune response. TGF-β inhibits early CRC while promoting metastasis, with butyrate-producing bacteria maintaining gut health, TMAO preventing protein denaturation, and SCF supporting microbiota homeostasis 5) Certain microbes like Enterotoxigenic *Bacteroides fragilis* and *Fusobacterium nucleatum* drive pro-tumorigenic inflammation by inducing IL-17 secretion from TH17 cells, promoting epithelial proliferation. This process recruits myeloid cells, such as polymorphonuclear neutrophils, which indirectly support tumor progression through ROS and RNS generation 6) Dysregulation of epithelial barrier pathways (MAPK, STAT3, NF-κB) disrupts colonic integrity. IL-23 triggers pro-inflammatory cytokine production, amplifying inflammation. TNF-α, IL-6, and IL-22 activate STAT3/NF-κB in transformed cells, promoting their survival and proliferation, impairing barrier function.

Microbiota can alter cancer susceptibility and progression through various mechanisms.^42^ In general, biofilm promotes cancer through various mechanisms. Firstly, they can induce inflammation that persists and fails to eliminate biofilm-associated pathogens. This chronic inflammation may lead to DNA damage, fostering the growth of cancer cells.^23^ Secondly, biofilms have the ability to modulate and impede the host immune response, creating an environment conducive to the development of cancer.^43,44^ Thirdly, certain bacteria within biofilms can produce toxins acting as carcinogens, thereby increasing the risk of cancer.^45^ Fourthly, bacteria residing in biofilms can alter host metabolism. Lastly, emerging evidence suggests that bacteria within the tumor microenvironment (TME), known as the tumor microbiome (TM), actively participate in cancer progression^46^ (Figure 2).In a recent study done by Cullin et al. demonstrated that cancer progression can induce changes in surrounding tissues, blood vessels, and immune responses, creating microenvironments conducive to the growth of specific bacterial strains.^47^

The study shows strong evidence that both bacterial biofilm formation and planktonic stage bacteria could contribute to cancer development. This association between bacterial biofilms and oncogenesis is unusual as it is based on molecular mechanisms.^48^

The human colon is protected by a thick mucus barrier primarily composed of mucins, especially Mucin 2 (MUC2). This barrier serves the crucial role of preventing direct contact between the colon’s epithelial cells and the microbiota. This mucus barrier is structured into two distinct layers. The inner layer, which is closely associated with the epithelial cells, is dense and effectively blocks the passage of bacteria, thus creating a separation between commensal bacteria and the host’s epithelial cells. Meanwhile, the outer layer, which is not firmly attached, serves as a natural habitat for commensal bacteria. This dual-layered mucus barrier enables the normal intestinal microbiota to reside within the colonic mucus without triggering an inflammatory response.^49^ When the protective mucus barrier is breached, it permits the microbiota to directly interact with the colonic epithelium. This event has been proposed as a crucial initial step that triggers alterations in the epithelial cells, ultimately leading to inflammation in the intestine.^50^ This heightened exposure of the colonic epithelium prompts adjustments in microbial interactions, consequently reshaping the microbial composition and function and frequently leading to the development of a biofilm.^40^ Bacteria can produce metabolites and signaling molecules that influence cancer cell proliferation, migration, and angiogenesis, ultimately fueling tumor progression.^51^ Additionally, biofilms have been found to enhance treatment resistance, making cancer cells less susceptible to chemotherapy, radiation therapy, and immunotherapy.^22,52^

In order to give readers a comprehensive picture of the state of knowledge about emerging implications of bacterial biofilm in different cancer biology at the moment, this review article focus on the recent advancements in the role of bacterial biofilm in cancer causation and outlook for the future. The most recent research results and clinical advancements, including screening techniques, molecular profiling, surgical intervention, targeted therapies, immunotherapy, and upcoming therapeutic options have been discussed in this review.

## Colorectal cancer

Colorectal cancer (CRC) ranks as the third most frequently diagnosed and the second deadliest cancer globally.^53^ In 2020, CRC accounted for around 9.4% of cancer-related fatalities.^1^ It was estimated that over 1.9 million new cases of CRC, including those affecting the anus, would emerge in 2020, resulting in approximately 935,000 deaths. It indicates that CRC comprised roughly one-tenth of all cancer incidents and deaths during 2020.^1^ In India, GLOBOCAN and the International Agency for Research on Cancer’s 2020 Indian fact sheets indicate the occurrence of more than 65,358 new cases of CRC and approximately 35,385 associated deaths.

Due to a significant rise in the number of reported cases among the elderly population, it is projected that the worldwide occurrence of CRC will more than double by the year 2035. CRC is a condition that exclusively affects the colon or rectum, and is characterized by the abnormal growth of glandular epithelial cells in the colon. There are three primary categories of CRC: sporadic, hereditary, and colitis-associated. The global incidence of CRC is steadily increasing, with both genetic and environmental factors contributing to an individual’s risk of developing this condition.^54,55^ Furthermore, as individuals with long-standing ulcerative colitis and Crohn’s disease grow older, their likelihood of developing CRC also rises.^56^

A well-balanced and healthy intestinal microbiota is essential for several key functions in the human body. It plays a critical role in extracting energy from food,^57^ shaping the structure of the intestinal lining,^58^ defending against harmful pathogens,^59^ and supporting the immune system.^60^ Contrary to common belief, when there is an imbalance in the intestinal microbiota, known as dysbiosis, it can disrupt various physiological processes in the host, ultimately contributing to the development of various diseases.^61^

In recent decades, there has been significant research into the comprehensive analysis of the microbiome associated with CRC. Among the bacteria found to play a significant role in promoting tumorigenesis, *F. nucleatum, pks+ E. coli, B. fragilis, E. faecalis, and Salmonella spp*.^62–64^ *Streptococcus bovis, Helicobacter pylori, and Clostridium septicum* ^38,65^(Table 1).

**Figure 3.**
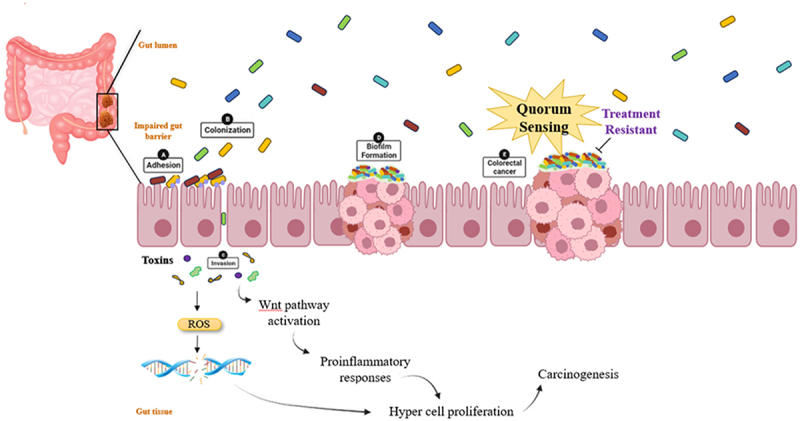
Role of bacteria and their metabolites in the causation and progression of CRC.

Trimethylamine-N-oxide (TMAO), a gut bacterial metabolite, is implicated in various diseases, including cardiovascular disease, liver cancer, diabetes, and colorectal cancer (CRC).^66,67^ TMAO’s connection to CRC involves its production from dietary sources by gut microbiota, influencing the tumor microenvironment and promoting tumorigenesis.^68,69^ It belongs to a class of microbiota-derived metabolites associated with CRC, impacting intestinal epithelial cells and immune responses.^70^ *E. coli*, typically a commensal organism, can become pathogenic with carcinogenic potential, particularly the *Pks+* strain. *Pks* island genes induce DNA double-strand breaks, promoting CRC development.^71,72^ Enterotoxigenic *Bacteroides fragilis* (ETBF) strains, producing *B. fragilis* toxin (bft), are linked to CRC, inducing tumorigenesis through immune signaling cascades and activation of pro-neoplastic changes.^73,74^ *F. nucleatum*, enriched in CRC tissue, promotes tumorigenesis through virulence factors and modulation of signaling pathways, contributing to primary cancer cell proliferation and metastasis^75,76^(Figure 3). *S. gallolyticus* subspecies *gallolyticus* (SGG) is associated with CRC, but its role as a primary driver is unclear. It may enhance tumor growth and colonization in mice, but further investigation is needed.^77,78^ *Salmonella enterica* infection, encompassing diverse bacteria, is linked to colon and gallbladder cancer development, with specific proteins, typhoid toxin, and AvrA, implicated in carcinogenesis through modulation of host immune responses and signaling pathways.^79,80^

However, there is an urgent need for CRC microbiome research to advance beyond descriptive studies. For example, organoids and gut-on-chip models can help to further microbiome research in a mechanistic way. A genotoxic ^*pks+*^*E*. coli mutational signature was recently found to be directly involved in CRC using the gut organoid model.^81^ It is evident that there is a complex relationship between the bacterial biofilm and CRC.An invasive biofilm shows pathologic features when it invades the mucous layer of the colon and directly contacts the epithelium.^82^ Around 50% of CRC patients and 13% of healthy people develop mucus-invasive bacterial biofilms on the colon mucosa.^83^

Research suggests bacteria-stimulated carcinogenesis shows how bacteria to bacteria and bacteria to hosts interact in CRC.^84,85^ Among the most crucial major pathophysiologic changes in the development of CRC in humans is the increase in the permeability of the gut and the enhancement of barrier activity losses brought on by the bacteria.^86^ These changes may be caused by biofilm. There are several likely mechanisms by which bacterial biofilms could worsen oncological conditions; in comparison to bacterial transformation, genotoxic stress caused by bacterial toxins has demonstrated the most substantiated links (Figure 3). For instance, a wide variety of bacteria produce several toxins,colibactin-synthesizing enzymes encoded by the pks+ pathogenicity island in *E. coli* induce DNA alkylation and double-strand breaks. Cytolethal Distending Toxin (CDT) from *E. coli* and *H. ducreyi* reduces replication fork speed in HeLa and U2OS cells, causing genetic instability. Enterotoxigenic *B. fragilis*, a source of gastrointestinal inflammation, induces the polyamine catabolic enzyme spermine oxidase (SMO), leading to an increase in ROS and DNA damage marker activation in colonic epithelial cells (HT29/c1 and T84), are implicated in genotoxicity and the onset of CRC.^81,87–89^

Increased tumor burden,^90^ increased metastasis,^91^ and chemo resistance^92^ are several indications of disease progression that have been linked to the enrichment of CRC-linked species in the gut microbiota. However, in CRC patients, there may be differences in the enrichment of taxa between fecal and mucosal samples. *F.nucleatum*, for instance, is frequently isolated from samples of biopsies of mucosal tissues rather than from fecal samples, despite having been previously related to CRC.^93^

Additionally, it has been found that CRC patient’s mucosal and fecal microbiomes have different metabolic outputs, which may be disease-relevant.^94^ Certain bacterial taxa can both enrich around colonic malignancies and produce intricate biofilms directly on lesions. Particularly in proximal colonic tumors, biofilms are commonly observed and are frequently associated with bacterial invasion of the tumor tissue itself.^40^ Direct interaction between bacteria and tumor cells may cause localized inflammation and the recruitment of immune cells,^95^ which eventually encourages an environment that is conducive to cancer.

## Gall bladder cancer

Gallbladder cancer (GBC) is the sixth most common and highly malignant cancer of the GI tract.^96^ Due to its deep location in the human body, GBC does not display any visible symptoms at the early stages, which results in poor diagnostic rates and a survival rate of less than five years due to poor prognosis.^97^ According to GLOBOCAN 2020 data, 115,949 newly diagnosed cases of GBC were reported and 84,695 deaths occurred in the year 2020.^1^ The formation of bacterial biofilms by *Salmonella typhi* in the gallbladder (GB) has been linked to an elevated susceptibility to the development of gallbladder cancer. *Salmonella typhi* is a Gram-negative, rod-shaped, flagellated and aerobic bacterium. Within the gallbladder, the antimicrobial activity of bile stimulates the production of exopolysaccharides containing O-antigens, facilitating the formation of *S. typhi* biofilms on gallstones.^97,98^ This biofilm growth is aided by the presence of bile and gallstones, which establish a favorable environment, supply essential nutrients for bacterial adhesion and growth on gallstone surfaces, and modulate the expression of proteins crucial for biofilm formation.^99^

S. typhi invades the mucosal surface of GB and forms a biofilm; releasing carcinogenic agents like bacterial glucuronidase, nitroso compounds,^100^ A2B5 typhoid toxin and cytolethal distending toxins (CDT) to promote GBC. CDT induces DNA damage, cell cycle arrest, and apoptosis.^101^ The CDT is a tripartite toxin complex composed of cdtA, cdtB, and cdtC polypeptides causes DNA damage, cell cycle arrest, and apoptosis.^102–104^

A2B5 typhoid toxin, causes DNA damage and G2 cell cycle arrest by binding exclusively to sialic acid-containing glycoproteins of host cells.^105^ This DNA damage activates various checkpoints and inactivates CDK1. Cells exposed to this toxin undergo senescence-associated beta-galactosidase activity, which aids the survival of cells accumulating DNA damage and causing genomic instability that may result in gallbladder cancer. Gallstone carriers demonstrate a higher *S. typhi* ratio compared to individuals with benign gallbladder diseases.^106,107^ As per a study, those who have *S. typhi* infection are more likely to get GBC, with a 22% positive rate.^28^

Numerous studies have highlighted the significance of bacterial effector proteins like SipA, *Salmonella* outer protein B SopB, *Salmonella* outer protein E SopE, *Salmonella* outer protein E2 SopE2, and SptP, which not only facilitate GB cell invasion but also activate mitogen-activated protein kinase (MAPK) and Protein kinase B (AKT) pathways, contributing to cell transformation observed in GC tumors of Indian patients infected by *S. typhi* .^108,109^ Biofilm formation, in *S. typhi* involves various contributing factors such as SPI-1 and SPI-2 genes encode type III secretion system which is essential for invasion and colonization,^110^ AsfD enhances *S. typhi* motility by elevating the expression of flagellar genes. Additionally, RibS upregulates cyclopropane fatty acids synthase gene (cfa), facilitating the conversion of unsaturated fatty acids to CFAs. This increased CFA content promotes bacterial biofilm production.^111,112^

Moreover, bile affects *S. typhi* biofilm formation by penetrating bacterial cells through Mig-14, an inner-membrane-associated protein. This protein enhances biofilm growth^113^ by altering the permeability of the outer membrane in response to adverse environments like the gallbladder acidic conditions and bile antimicrobial nature. GalE contributes to biofilm formation through the production of uridine diphosphogalactose-4-epimerase, vital for the formation of lipopolysaccharide O-antigen of *S. typhi*. The GalE mutant (Tn10 inserted) containing *S. typhi* developed a very thin biofilm on gallstones.^99^

Quorum sensing (QS) emerges as a pivotal mechanism in the intricate interplay between *S. typhi* and the gallbladder environment, adding another layer of complexity to the development of gallbladder cancer (GBC). In stressful conditions, QS is a key player in biofilm formation, and *S.* *typhi* utilizes two autoinducers (AI-I and AI-II). Specifically, AI-II, generated through the luxS synthase gene, governs the expression of virulence genes in bacterial pathogens. A luxS gene mutant exhibited reduced biofilm-forming ability,^114^ leading to downregulation of chemotaxis genes, spi genes, and motility-related genes.^115^ The intricate interplay between *S. typhi* and the gallbladder environment has been shown to contribute significantly to the development of GBC. These findings highlight the importance of addressing bacterial infections and biofilm formation as potential factors in the prevention and management of gallbladder cancer.

## Head and neck cancer

Head and neck squamous cell carcinoma (HNSCC) rank as the sixth most prevalent cancer worldwide, encompassing various subtypes such as oral, pharyngeal, sinus, nasal cavity, and salivary gland carcinoma. Notably, oral cancer is primarily associated with biofilm formation. The primary risk factors for head and neck cancer include cigarette smoking and alcohol consumption, environment plays a crucial role in shaping the bacterial community, influenced by factors such as temperature, oxygen tension, pH, substratum properties, nutrient availability, and exposure to cell and immune signaling.^116,117^ In the context of HNSCC, poor oral hygiene may contribute to microbial overgrowth or alter the composition of the oral microbiota, leading to immune homeostasis disruption. Inflammation, as a consequence, can further alter the types of microbes in the oral niche, perpetuating dysbiosis. Bacterial presence in HNSCC tissues has been confirmed through both microbiological culture and 16S rRNA sequencing.^118^ This intricate interplay between dysbiosis of the microbiome and the formation of biofilms significantly influences the development of HNSCC.^119^

Moreover, the oral microbiome is directly associated with the carcinogenicity of these substances by producing mutagenic acetaldehyde from alcohol.^120^ An intriguing aspect is the potential of bacterial biofilm growth in epithelium to create conditions leading to oncogenic transformation of epithelial cells. This phenomenon has been demonstrated in the context of colon carcinoma.^40^ Biofilm formation causes significant disturbances in inflammatory metabolites, induces epithelial damage, and promotes cell proliferation.^121^ Moreover, it has been shown to induce apoptotic processes in the head and neck region.^122^ The association between bacterial biofilms and their impact on the local environment sheds light on additional mechanisms contributing to the development and progression of HNSCC. Recent research has unveiled distinctive microbiomes within oral HNSCC, characterized by the presence of *Selenomonas*, *Fusobacterium*, *Leptotrichia*, and *Treponema*. Conversely, non-oral HNSCC tends to exhibit a higher prevalence of *Clostridium* and *Pseudoalteromonas* .^123^ These intriguing discoveries emphasize the utmost significance of understanding the intricate interplay between microbial communities and biofilms during the initiation and progression of HNSCC. This knowledge opens up promising pathways for pioneering breakthroughs in combatting this widespread and formidable type of cancer.

## Oral cancer

Oral squamous cell carcinoma (OSCC) is the most prevalent malignant cancer of the oral cavity. OSCC stands as the most prevalent subset of head and neck cancer, contributing significant morbidity and mortality worldwide. Recent studies have confirmed that periodontitis triggers microbial dysbiosis and virulence factor expression, rather than introducing new pathogens.^124,125^ Within a study it was found that within the oral microbiome of individuals with OSCC, *Fusobacteria* played a pivotal role in the upregulation of virulence factors such as capsule biosynthesis, flagellum synthesis and assembly, chemotaxis, iron transport, hemolysins and adhesins.^126^ *F. nucleatum* due to its rod shape serves as a crucial bridge between initial colonizers like *Streptococcal spp* and later inhabitants such as *Porphyromonas gingivalis*.^127^ This vital role contributes to the intricate web of polymicrobial biofilms and facilitates dynamic interactions among microorganisms.^128^ In an investigation, a notable increase in the abundance of *Porphyromonas* and *Fusobacterium* was evident in OSCC tissues compared to normal tissue counterparts.^129^ In OSCC samples, *F. nucleatum* was the dominant species,^130^ with *Pseudomonas aeruginosa* being the second most common.^131^ Similarly, among the three *Fusobacterium* species, *F. nucleatum* was significantly elevated in oral cancer, while *P. gingivalis* remained consistent across groups.^132^

*F. nucleatum* has been associated with initiating oral malignancy in a chemically induced mouse model of OSCC.^133^ This study revealed that both *P. gingivalis* and *F. nucleatum* have the ability to induce carcinogenesis by direct interaction with Toll-like receptors (TLR) of oral epithelial cells through FadA and upregulates IL-6-STAT3 signaling pathway. Furthermore, *F. nucleatum* infection promotes cyclin D1 and matrix metalloproteinase-9 (MMP-9) activity, influencing the progression and invasiveness of oral tumors.

According to “two-hit” theory somatic alterations serves as the initial hit and *F. nucleatum* serves as the second hit, intensifying cancer progression from benign to malignancy.^134^ *F. nucleatum* triggers genomic instability, maintains growth signals, suppresses tumor suppressor genes, weakens immune responses, promotes pro-tumor inflammation, stimulates invasion and metastasis through epithelial-mesenchymal transition (EMT).^35^ Further studies are needed to completely understand how *F. nucleatum* contributes to OSCC development at the molecular and cellular level.

## Pharyngeal cancer

Oral cavity and pharynx neoplasms rank as the seventh most prevalent cancers globally, with around 710,000 new cases annually and 359,000 deaths per year.^135^ While smoking and alcohol consumption are widely recognized as major risk factors for these cancers, the International Agency for Research on Cancer has acknowledged certain HPV genotypes as carcinogens for specific head and neck anatomical subsites since 2007. Among the 200 types of HPV, approximately 15 are linked to malignancies, with HPV16 standing out as one of the most common and potent carcinogenic types.^136^ Although the exact contribution of biofilms to the development and advancement of this cancer remains a subject of ongoing investigation, interesting findings have been found in patients with nasopharyngeal carcinoma (NPC) who have undergone radiotherapy. These individuals have reported side effects, including osteoradionecrosis (ORN) and mucositis. Oral mucositis is a prevalent condition characterized by acute inflammation of the oral mucosa, often occurring as a complication of systemic cancer therapy or radiotherapy. It is particularly common following radiotherapy for nasopharyngeal carcinoma (NPC),which have been associated with the development of biofilms.

Radiotherapy for head and neck cancer (HNC) can cause significant harm to salivary glands and oral mucous membranes, leading to reduced saliva production and a compromised epithelium. This creates favorable conditions for bacterial growth in the oral cavity.^137^ Recent research indicates a crucial role of the oral microbiota in osteoradionecrosis (ORN) development,^138^ with increased *Actinomycetes* abundance^139^and prevalence of *Streptococcus intermedius* in irradiated patients.^140^

Li et al. study found that osteoradionecrosis (ORN) lesions exhibit changes in oral microflora compared to healthy tissues, with increased abundance of bacteria such as *Prevotellaceae, Fusobacteriaceae, Porphyromonadaceae, Actinomycetaceae, Staphylococcaceae, Prevotella, Staphylococcus*, and specific species like *Endodontalis* and *Prevotella intermedia*. Conversely, *Streptococcaceae, Streptococcus*, and *Haemophilus* were more abundant in normal tissues.^141^

*Prevotella* and *Streptococcus* were hypothesized to play functional roles in ORN lesions and healthy oral tissues, respectively, and identified as potential diagnostic and prognostic biomarkers. *Prevotella*, associated with ORN progression, stimulates dendritic cells via toll-like receptor 2, leading to IL-1β, IL-6, and IL-23 release. This triggers T helper 17 cell-mediated IL-17 production, activating neutrophils and promoting periodontitis.^38^ Radiation exposure transforms normally commensal *Prevotella* into an opportunistic pathogen, producing virulence factors. *Prevotella*-induced lipopolysaccharides (LPS)^39^ inhibit osteogenesis and induce osteoclast formation, potentially impairing bone regeneration. Enriched biosynthesis and metabolism-related pathways in ORN microorganisms suggest *Prevotella’s* complex role beyond contamination, contributing to bone loss. *Prevotella* emerges as a potential prognostic biomarker for ORN clinical progression.^141^ A noteworthy study revealed a higher prevalence of biofilms in NPC patients who developed ORN compared to their counterparts who did not exhibit ORN.^142^

These biofilms possess unique compositions, featuring *methicillin-resistant Staphylococcus aureus* and *Streptococcus viridans* in ORN biofilms. *Staphylococcus aureus* typically resides as a component of the body’s normal flora. However, extensive antibiotic use has led to the development of methicillin-resistant strains (MRSA). These MRSA variants have the ability to create biofilms and are associated with various infections, including those involving indwelling foreign bodies, bacteremia, soft tissues, endocardium, and bones such as osteomyelitis. ^143–145^ In the context of mucositis, *Actinobacteria*, *Veillonella*, and *Fusobacteria* are prominent. Interestingly, *Veillonella* plays a pivotal role in facilitating the adhesion of *Streptococcus mutans*, while *Actinobacteria* disrupt amino acid metabolism, leading to a slowdown in the healing of mucosal injuries caused by radiotherapy.^146^

In addition, recent research revealed *Streptococcus* (37.3%), *Fusobacterium* (11.3%), and *Prevotella* (10.6%) as the dominant genera in the swab and tissue samples of pharyngeal carcinoma patients.^147^ These biofilms exhibit resistance to conventional treatments for pharyngeal cancer, presenting a profound challenge in effective management. The characteristics of biofilms, such as limited nutrient diffusion, constrained antimicrobial passage, and modification of the surroundings to create a less favorable environment, collectively result in extensive resistance and tolerance to antimicrobial agents. The intricate relationship between biofilms and pharyngeal cancer continues to evolve, necessitating further investigation to fully unveil their specific contributions and mechanisms. Nevertheless, the presence of biofilms and their impact on treatment-related side effects in patients with pharyngeal cancer after radiotherapy remain an intriguing and vital area of scientific interest.

## Salivary gland carcinoma

Salivary gland carcinoma (SGC) is the cancer of salivary glands and it is the rare type of head and neck carcinoma. Incidence ranging from 0.4 to 3.5 per 100,000 per year in the Western world. The role of biofilm in the development of SGC is not fully explored. Some studies have proposed a potential association between *Helicobacter pylori* and salivary gland tumors. Notably, a study uncovered an intriguing connection between localized *H. pylori* infection, Sjögren’s disease, and salivary gland MALT lymphoma.^37^ Interestingly, *H. pylori* seems to act as an additional antigenic stimulation, influencing the development of both salivary gland and gastric MALT lymphoma. This discovery implies that *H. pylori* may serve as a booster, promoting B cell proliferation and contributing to the progression of lymphoma. In research done by Jiang et al. unstimulated saliva samples were collected from 13 SACC patients and 10 healthy controls.^36^ Through comprehensive analysis using 16S rRNA sequencing and whole-genome shotgun metagenomic sequencing, the study examined microbial diversities, compositions, and functions. The alpha diversity analysis revealed no significant differences between SACC patients and healthy controls. However, beta diversity demonstrated a discernible separation trend. SACC patients exhibited higher levels of *Streptococcus* and *Rothia*, while *Prevotella* and *Alloprevotella* were more prevalent in healthy controls. This research sheds light on the microbial landscape in the oral microbiota of individuals with SACC and highlights distinct differences in microbial abundances between SACC patients and their healthy counterparts.

However, the precise mechanisms, relationships between biofilms and salivary gland cancer remain a subject of ongoing investigation and scientific debate.

## Lung cancer

Lung cancer (LC) ranks as the second most frequently diagnosed cancer in both males and females, trailing prostate and breast cancer. In 2020, LC affected 2.2 million individuals, which accounted for 11.4% of all cancers. This disease resulted in 1.7 million deaths, making up around 18% of all cancer-related deaths globally (Globocan, 2020). While cigarette smoking stands as the one of the predominant causes of lung cancer,^148^ recent studies have unveiled a compelling connection between chronic pulmonary infections and the development of LC. Interestingly, such infections may act independently or in conjunction with smoking, elevating the risk of lung cancer.^149,150^ Tissue samples from lung cancer patients have uncovered the presence of diverse pathogens, with *mycoplasma* being prevalent in lung carcinomas.^151^ Studies suggest that *mycoplasma*-infected cells (Microplasma arginine infected RPMI 4788 cells) *invivo* exhibit potential for cancer metastasis compared to non-infected cells.^152^

Moreover, several meta-analyzes have shown an increased susceptibility to lung cancer among individuals with active pulmonary tuberculosis. It is important to highlight that standard TB treatment lasts for 6 to 9 months, during which the significant lung inflammation resulting from TB infection may play a role in a chronic inflammatory process associated with cancer development.^153,154^

In addition to *Mycoplasma* and *Mycobacterium tuberculosis*, certain bacteria, such as *Chlamydia pneumoniae* and *Staphylococcus strains*, have emerged as potential contributors to lung carcinogenesis. ^155–157^ Some studies also found bacterial colonies in the respiratory tracts of lung cancer patients, including *Haemophilus influenza* and *Candida albicans*, which are particularly linked to lower respiratory tract malignancies. Furthermore, *Legionella pneumophila*, along with strains of *Bacillus*, *Listeria*, and *Streptococcus*, have been detected in lung cancer patients.^158,159^ The available data strongly supports the intriguing idea that lung cancer may be linked to chronic infections involving biofilms. As a result, a growing number of researchers are suggesting that lung malignancies might essentially be communities of various pathogens that have developed resistance to antibiotics. This emerging perspective underscores the potential role of persistent bacterial infections in lung cancer, prompting the exploration of novel strategies to understand these intricate interactions more effectively.

## Gastric cancer

Gastric cancer (GC) remains a significant worldwide health concern, making a substantial impact on cancer-related fatalities. It ranks as the fifth-most prevalent cancer worldwide.GC new cases: 1,089,103 (5.6%) and caused approximately 768,793 (7.7%) deaths in 2020.^160,161^ Current research has revealed the role of bacterial biofilms in this intricate disease process, even though *Helicobacter pylori* infection has long been linked to the development of GC.^162^ The incidence and mortality rates for GC in Eastern Asian countries are notably higher than in other regions, accounting for more than 60% of global cases.^162^ In many localized chronic infections, the formation of biofilms by bacterial populations is the key virulence factor.^163^ There are several risk factors linked to GC, including *Helicobacter pylori* infection, genetic changes, ethnicity, dietary habits, and lifestyle choices.^164,165^ Additionally, specific inherited GC syndromes, like hereditary diffuse gastric cancer (HDGC) resulting from mutations in the CDH1 tumor suppressor gene, are associated with a significantly elevated risk of GC. CDH1 mutations are believed to be responsible for GC development in approximately 80% of patients.^164^

The initial proof of *H. pylori* ability to form biofilms while establishing itself in the human gastric mucosa was captured through photographic documentation.^166^ In the context of GC, the bacterial biofilm comprises various species, with *H. pylori* often serving as the cornerstone organism. Other bacterial species, such as *Streptococcus, Prevotella, and Fusobacterium*, have also been found to be associated with GC biofilms^167,168^ In the human, gastric lumen is known for its harsh conditions, which are detrimental to many bacteria. Despite these challenges, *H. pylori* manages to thrive by utilizing its urease activity to counteract and neutralize the acidity.^169^

On the other hand, biofilm development might be more important for its ongoing colonization. Surprisingly little is understood about the development of biofilms on human gastric mucosa while they are in-vivo. Using powerful electron microscopes, various research studies revealed dense clusters of *H. pylori* .^170,171^ While *H. pylori* isolated from GC patients is usually non-culturable, other methods (PCR or histological method) can detect them; as well, *coccoid* forms are more frequent in gastric mucosa of GC patients than those of peptic ulcer patients.^172^

Recent studies utilizing transcriptomic and proteomic analyzes have obtained significant insights into the shift from the planktonic phase to the biofilm stage in *H. pylor*i. These investigations reveal increased gene expression related to adhesins, flagella, toxins, efflux pumps, lipopolysaccharides (LPS), the type IV secretion system, urease, and hydrogenase. Such heightened expression suggests adaptations for acquiring alternative energy sources during the transition^173^ and during prolonged inflammation plays a role in the development of several malignancies, but it is especially crucial for GC linked to *H. pylori* .^174^ Certainly, when gastric mucosa inflammation persists, it leads to elevated nitric oxide production. This, in turn, plays a role in causing harm to nucleotide bases and influencing transcriptional regulation by boosting DNA methyl transferase activity.^175^ Indeed, for the E-cadherin tumor suppressor gene, its promoter region has often been seen to be hypermethylated in *H. pylori* infections of adult patients, and its transcriptional activity will be altered in gastric cells.^176^ Inflammatory responses have been shown to be controlled by bacterial virulence factors. *H. pylori* virulence factors, such as the VacA and CagA, can inhibit T-cell activation and evade recognition by Toll-like receptor. Additionally, CagA has been shown to promote epithelial to mesenchymal transition (EMT), which contributes to the generation of cancer stem cells.^177,178^ On the other hand, γGT can promote oxidative stress and enhance apoptosis of gastric cells.^174^ Inherent host-specific mechanisms counterbalance these responses. Evidence from knockout animal models underscores the significance of host factors in regulating inflammation. For instance, the anti-inflammatory outcomes linked to TLR9 signaling highlight the role of host elements in modulating inflammatory processes.^174^

There are various factors that might account for the presence of bacterial biofilms in stomach cancer. The primary advantage of biofilms is that they give bacteria a protective habitat from various immunological reactions and antibacterial substances. Infection and chronic inflammation may result from this enhanced resilience, which is a known risk factor for GC. Inflammation of the gastric mucosa can be triggered by bacterial biofilms.^23^ The sustained inflammation may cause DNA damage and genomic instability, both precursors of cancer initiation, over time due to the production of reactive oxygen and nitrogen species.^179,180^ Recent studies suggest that bacterial biofilms can affect epithelial-mesenchymal transitions (EMTs).It may be contributing to cancer metastasis that EMT is induced by biofilms.^181,182^ The expression of microRNAs, which are short, non-coding RNAs that control gene expression, can be altered by bacterial biofilms. Alterations in microRNA expression may encourage oncogenic transformations in gastric epithelial cells, promoting the growth and development of tumors.^183,184^

## Pancreatic cancer

Pancreatic cancer (PC) is a highly aggressive malignancy, the incidence and mortality rates of pancreatic cancer are closely related.^185^ In 2020, it ranked as the 14th most prevalent cancer type, with approximately 495,733 newly diagnosed cases and 466,003 reported fatalities.^1^ There is a 50% survival rate after 6 months for patients diagnosed with cancer. Currently, pancreatic cancer patients with chemotherapeutic options live nearly as long as their incidence because chemotherapy only marginally prolongs life.^186^

Before 2030, PC is predicted to overtake all other cancers as the second leading cause of death.^187^ PC incidence rates each year are rapidly rising. Up to 90% of PC patients pass away within five years of diagnosis, and more than 50% die so within the first six months.^188,189^ Pancreatic cancer risk has recently been associated with bacterial infections that lead to periodontal disease^190^

There have been a number of studies linking *P. gingivalis* and *F. nucleatum* to PC.^191,192^ Furthermore, Wei et al. noted that both *Leptotrichia* and *Streptococcus* were associated with a higher risk of developing PC.^30^ Pushalkar et al. observed an increase in the relative abundance of *Proteobacteria, Synergistetes*, and *Euryarchaeota* in the fecal samples of individuals with pancreatic ductal adenocarcinoma (PDAC) compared to healthy controls.^193^ Additionally, a small study by Half et al. reported elevated levels of *Sutterela, Bacteroides, Odoribacter, and Akkermansia* in the feces of patients with pancreatic cancer (PC) compared to healthy individuals.^194^ Another study done by Half et al. found reduced levels of *Firmicutes* genera in the fecal samples of PC patients. Examining overall gut microbial diversity,^195^ Ren et al. observed a significant reduction in diversity among PC patients, though no significant differences were identified between different PC subtypes.^196^ Thomas et al. using a murine model, demonstrated that the intestinal microbiota play a crucial role in pancreatic cancer progression, as mice with depleted microbiota exhibited decreased tumorigenicity.^197,198^ Emphasized that alterations in fecal microbiota composition manifest early in the course of tumor progression in a murine model of PDAC.Gut dysbiosis is implicated in pancreatic cancer development by activating chronic inflammation. LPS, through Toll-like receptor 4 (TLR4), inhibits tumor suppressor proteins (PTEN, pRb, MAP2K4, p53) and induces HIF-1α and STAT3, promoting cell migration and epithelial – mesenchymal transition (EMT)),^199–201^ Additionally, LPS, interacting with NF-κB, MyD88, and AKT, upregulates programmed cell death ligand 1 (PD-L1), reducing immune responses by inducing apoptosis of tumor-infiltrating lymphocytes (TILs).^202^ While LPS may lead to long-term inflammatory cell depletion, in early stages, it increases local CD3+ and CD8+ T cells. The gut microbiota contributes to pancreatic cancer by inducing sustained inflammatory responses with the production of reactive oxygen species (ROS) and reactive nitrogen species (RNS).^203^ ROS have the potential to harm DNA and cell membranes, while also disrupting proper protein folding and increasing concentrations of oncogenes. An alternative suggested mechanism entails communication between gut microbiota and pancreatic cells via the mammalian target of the rapamycin (mTOR) pathway. This interplay affects cellular processes such as growth, autophagy, and cytoskeletal organization. This interaction influences cell growth, autophagy, and cytoskeletal organization.^204^ Chronic inflammation, elevated oncogenes (e.g., *Kras*), and microbiota-induced barrier disruption collectively contribute to pancreatic carcinogenesis.^205,206^ There is strong evidence that *P. gingivalis* promotes the progression of pancreatic cancer, which has been validated in murine models.^207,208^ PC may be associated with bacterial biofilms.In spite of the limited knowledge of biofilm formation in the pancreatic ducts,^25^ no direct link has been established between the PC and bacterial biofilms.^20^

## Discussion

Bacterial biofilms have significant clinical implications, as they highlight the potential need to address as a therapeutic target to improve treatment efficacy. Moreover, the complex interplay between bacterial biofilm, and the immune system within the tumor microenvironment can dictate the overall prognosis for cancer patients.^209^ Certain bacterial species within the biofilm have been associated with a pro-tumorigenic immune response, leading to poor clinical outcomes. Conversely, some bacteria have shown immune-stimulatory properties, eliciting an anti-tumor immune response.^210^

Understanding these intricate dynamics may provide opportunities for personalized therapies that target the tumor-associated microbiota and optimize treatment strategies.^211^ In this era of precision medicine; the recognition of bacterial biofilms as influential players in cancer biology adds a new layer of complexity to our understanding of the disease. The mechanisms by which biofilms impact tumorigenesis, metastasis, and treatment response is an active area of research. By deciphering these connections, researcher and clinicians can identify novel therapeutic targets and develop innovative strategies to improve cancer treatment outcomes. The emerging association of bacterial biofilms in cancer biology have opened up a new frontier in cancer research.^212^ Understanding the role of biofilms within the tumor microenvironment holds promise for advancing our knowledge of cancer progression, treatment resistance, and patient outcomes. By investigating and targeting these microbial communities, these findings may pave the way for innovative approaches that can augment existing cancer therapies and ultimately improve the lives of cancer patients worldwide.
